# Endocrine disrupting chemicals and the adrenal gland

**DOI:** 10.55730/1300-0144.6121

**Published:** 2025-10-13

**Authors:** Gülay ŞİMŞEK BAĞIR, Melek Eda ERTÖRER

**Affiliations:** Department of Endocrinology and Metabolism, Faculty of Medicine, Başkent University Adana Dr. Turgut Noyan Application and Research Center, Adana, Turkiye

**Keywords:** Endocrine disruption, adrenal, hormones

## Abstract

Endocrine disrupting chemicals (EDCs) are exogenous compounds that have hormone-like effects inside the body. Endocrine disruptors can affect adrenal glands in humans and animals. Adrenocortical dysfunction related to chemical exposure has been reported in the literature.

The human adrenal gland is crucial for the synthesis of steroid hormones (glucocorticoids, mineralocorticoids, and androgens) and amino acid-derived hormones (epinephrine and norepinephrine). All steps in the adrenocortical steroidogenesis pathway are potential targets for chemical inhibition, including the adrenocorticotropic hormone receptor, steroidogenic acute regulatory protein, mitochondrial cytochrome P450 (CYP) enzymes (CYP11A1, CYP17, CYP21, CYP11B1, and CYP11B2), and 3-hydroxysteroid dehydrogenase D4,5 isomerase. EDCs either inhibit the enzymes in steroid biosynthesis or, occasionally, activate them further.

Several studies have reported evidence linking EDC exposure to different cancers, early puberty, and reproductive dysfunction. The risk of these harmful effects is higher during development. Pregnant women, babies, children, and adolescents are especially vulnerable, and should therefore be kept away from these chemicals.

## 1. Introduction

Endocrine disrupting chemicals (EDCs) are exogenous substances found in soil, water, and food. These chemicals can be detected in human and wildlife tissues. When ingested into the body, they have hormone-like effects and disrupt endogenous regulation. Plant-based hormone-like substances naturally occur in foods, while industrial environmental pollutants such as dichlorodiphenyltrichloroethane (DDT), polychlorinated biphenyls (PCB), bisphenol A (BPA), polybromide diphenyl ethers, and phthalates [1,2] are also examples of EDCs. Scientific investigation into the role of EDCs on the functional impairment of endocrine glands, such as the pituitary, thyroid, and adrenal glands, has intensified in recent years [3–5]. The exact mechanisms of action involved in EDC toxicity are not fully understood. However, numerous mechanisms are thought to be involved, including oxidative stress, genotoxic and epigenetic effects, interaction with nuclear receptors, and increased endogenous hormone sensitivity. The possible effects of EDC exposure include various endocrine and immune system diseases, different cancers at younger ages, early puberty, and reproductive dysfunction [1,2]. The risk of these harmful effects is higher during development. For example, chemical exposure of the fetus during pregnancy can adversely affect fetal development [6]. Therefore, vulnerable groups such as pregnant women, babies, children, and adolescents should be kept away from these chemicals.

## 2. Endocrine disruptors and the adrenal gland

### 2.1. Features of the adrenal gland that cause vulnerability to EDCs

**Figure 1:**
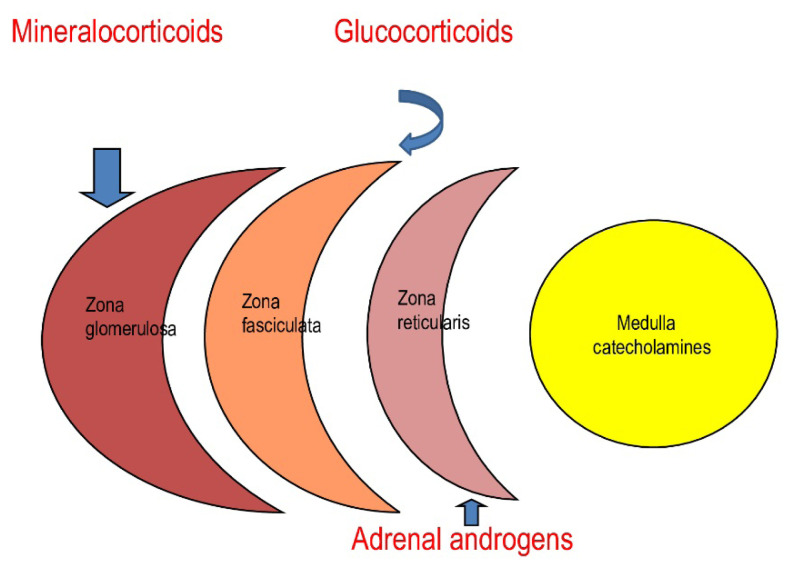
Illustration representing a cross-section of an adrenal gland.

**Figure 2:**
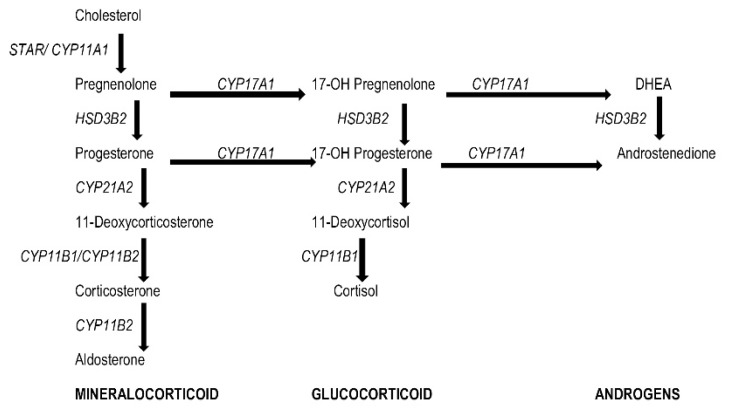
Adrenal steroidogenic pathways. Adapted from Williams Textbook of Endocrinology, Adrenal Cortex and Endocrine Hypertension, Chapter 13, page 476. StAR: steroidogenic acute regulatory protein, CYP: mitochondrial cytochrome P450 type, CYP11A1: P450 side-chain cleavage enzyme, CYP11B1: 11β-hydroxylase, CYP11B2: aldosterone synthase, CYP17A1: 17α-hydroxylase, CYP21A2: 21-hydroxylase, DHEA: dehydroepiandrosterone, DHEAS: dehydroepiandrosterone sulfate, HSD3B2: 3β-hydroxysteroid dehydrogenase type 2.

Adrenal glands are intraabdominally located, bilateral vital organs. Each gland is composed of 2 main anatomical regions: the adrenal cortex and adrenal medulla. The latter lies at the center of the gland surrounded by the cortex. Although the two parts secrete different hormones, they interact due to their close proximity. The adrenal medulla secretes catecholamines—amino acid-derived hormones that include epinephrine and norepinephrine—and fractionated metanephrines. These hormones are responsible for maintaining sympathetic activity and vascular tone. The adrenal cortex is composed of 3 regions. From outside to inside, these are the zona glomerulosa, zona fasciculata, and zona reticularis (Figure 1). Each region secretes different steroid hormones, including mineralocorticoids (mainly aldosterone), glucocorticoids, and adrenal androgens, respectively. Oversecretion of adrenal hormones may result in serious health problems, whereas adrenocortical impairment can lead to life-threatening adrenal crisis and death. Enzymatic pathways of the adrenal cortex are shown in Figure 2 [7].

Adrenal glands play a pivotal role in every kind of stress response. For instance, administration of drugs and chemicals at toxic doses results in the activation of the hypothalamo–pituitary–adrenal (HPA) axis and adrenocorticotropic hormone (ACTH) secretion is stimulated. Thus, stimulated adrenals increase in size and weight, and both zona fasciculata thicken microscopically. As glucocorticoid secretion increases, a rich blood supply to and from the gland results in rapid distribution of steroids, even to distant parts of the body. HPA activation is an adaptation for survival, enabling the organism to cope with physiologically and metabolically adverse situations. However, these features also make the glands vulnerable to toxic insults. The rich vascular supply can also bring large amounts of toxic substances to the adrenals. Lipophilic milieu due to high content of cholesterol esters in the adrenal glands facilitates deposition of lipophilic toxicants. Lipophilic nature, cholesterol its main content, facilitates deposition of lipophilic compounds within the glands. Moreover, adrenals are prone to damage by lipid peroxidation through metabolites and free radicals because of a high unsaturated fatty acid content in adrenocortical cell membranes [8].

### 2.2. Studies on EDCs and the adrenal gland

EDCs can affect all endocrine glands in both humans and animals. Organisms are simultaneously exposed to hundreds of different EDCs at various doses. It is therefore not usually possible to detect the exact EDC and its toxic dose for a given disorder. In addition, it is not clear whether a single EDC or a cocktail of EDCs is responsible for a given disorder. Thus, the relevant literature is based on observational studies of wildlife, in vitro cell lines, or in vivo animal studies.

Evidence of adrenocortical dysfunction related to EDC exposure has been found in birds, fish, and marine mammals. Disrupted cortisol production as a result of the Deepwater Horizon oil spill has been reported in marine organisms. Abnormal aldosterone production due to exposure to pollution has also been reported in dolphins [9,10]. Chemical residues that enter food chains are important sources of EDCs for wildlife.

The response of various receptors, tissues, and organs varies depending on the type of EDC. These chemicals either inhibit the enzymes in steroid biosynthesis or, occasionally, activate them further. Some enzymes of the cytochrome P450 (CYP) family may play a role in the bioactivation of 7,12-dimethylbenz[a]anthracene [11]. The adrenal cortex stores lipoproteins as esterified lipids after receptor-mediated uptake. Accordingly, animal studies have shown that adrenal cells can capture numerous toxic agents including DDT metabolites, methacrylonitrile, and PCB metabolites, and transport them inside the cell [12–14].

Various pharmaceutical medicines and industrial environmental pollutants that can affect different steps in the steroid biosynthesis pathway have been detected in adrenal glands (Table). All steps of adrenocortical steroidogenesis are potential targets for chemical inhibition [8]. This includes the ACTH receptor, steroidogenic acute regulatory protein (StAR), CYP enzymes (CYP11A1, CYP17, CYP21, CYP11B1, CYP11B2), and 3-hydroxysteroid dehydrogenase D4,5 isomerase.

Several studies have shown that chemicals such as pesticides, plasticizers, dioxins, PCBs, and polycyclic aromatic hydrocarbons can affect adrenal glands in vitro and in vivo. For example, BPA targets hydroxysteroid dehydrogenase enzymes, while phthalates, chlorinated phenols, and some phytoestrogens inhibit sulfotransferases [3]. During steroid hydroxylation reactions, reactive oxygen species cause oxidative stress. BPA can inhibit antioxidant enzymes such as superoxide dismutase, catalase, glutathione reductase, and glutathione peroxidase [15]. PCBs target and impair CYP17, CYP21, CYP11B1, CYP11B2, CYP19 (aromatase), and dehydrogenases (3-hydroxysteroid dehydrogenase D4,5 isomerase and 17b-hydroxysteroid dehydrogenase) [8]. Fatal adrenocortical toxicity of pharmaceutical medicines, aminoglutethimide, and etomidate is well known in humans. Toxicological inhibition of adrenocortical steroidogenesis causes various hormonal deficiencies, as cortisol and aldosterone synthesis pathways are also negatively affected. While aminoglutethimide affects ACTH receptors, CYP11A1, and CYP11B1, etomidate affects CYP11B1 [8,16,17]. Patients who are prescribed these medications should be monitored carefully for life-threatening adrenal toxicity. Polybrominated biphenyls, 2,3,7,8-tetrabromodibenzo-p-dioxin, tetrabromobisphenol-A, triazines, atrazine, simazine, propazine, ditributyl and phenyltin chlorides, flavonoids, and bromophenols are chemicals that target the steroidogenesis pathway and cause adrenocortical toxicity [18–20]. Toxic doses of the pesticide DDT can cause cell atrophy and degeneration in the zona fasciculata and zona reticularis. Rat studies have shown that exposure of chronic nontoxic doses of DDT can cause deterioration in morphogenesis of the adrenal cortex and medulla, consequently disrupting hormone secretion in the adrenal cortex and chromaffin cells. All 3 adrenocortical zones are affected by the disruptive effect of DDT. For instance, the zona glomerulosa and zona reticularis are very sensitive to low and high doses of DDT, while the zona fasciculata is less affected by nontoxic, low doses of DDT [1]. Parabens are commonly used for preservation of various foods, cosmetics, and pharmaceutical products. They can easily be absorbed by the human body. Animal studies with butyl paraben (BuP) have shown that BuP exposure can decrease *StAR* gene expression in female fetuses; however, conflicting articles also exist in literature [21,22].

The H295R cell line is often used for in vitro studies that examine the impact of chemicals on adrenocortical steroidogenesis. This cell line is produced from human adrenocortical carcinoma cells and produces both aldosterone and cortisol. In vitro studies provide an insight into the expression of genes and specific enzyme domains in response to chemical exposure [23,24].

Low dose EDC exposure can cause subclinical or latent long-term dysfunction in endocrine glands. Adrenal glands can act as reservoirs of toxic metabolites and free radicals because of their rich blood supply and lipophilic cell membranes. Accordingly, Fommei et al. [25] detected more α-, β-, and γ-hexachlorocyclohexane, hexachlorobenzene, and PCB in aldosterone-producing adenomas compared to normal cortex. This topic needs further investigation [25,26].

Of all endocrine organs, toxicological studies show that adrenal glands are the most affected by drugs and chemicals, mainly because of their central role in responding to physiological stress [27]. Maximum tolerable doses of drugs or chemicals are stressful for organisms, activating the HPA axis resulting in enlargement of the adrenal glands. Fundamentally, the stress response to environmental waste is a survival mechanism. Changes in size and weight of adrenals are generally dependent on the effect of chemicals on ACTH activity. For instance, agents that contain glucocorticoid agonists act like endogenous steroids and cause negative feedback inhibition on the hypothalamus and pituitary. Because of decreased ACTH secretion, these inhibitory effects result in marked thinning of the zona fasciculata and subsequent adrenal atrophy. Conversely, drugs and chemicals that inhibit the adrenocortical steroidogenesis pathway can block glucocorticoid production and increase ACTH secretion as they eliminate the negative feedback on the HPA axis. Accordingly, cyanoketonea, a potent adrenocortical enzyme inhibitor, can cause a 100% increase in adrenal weight in 3 days by affecting 3-hydroxysteroid dehydrogenase D4,5 isomerase [28]. BPA exposure increases adrenal weight and disrupts the stress response in rats [29]. In utero phthalate exposure can cause a permanent decrease in major steroid hormone synthesis in the adrenal tissue of male rats [30].

Exposure to EDCs via breathing in air pollution is a serious health problem related to industrialization. Inhalation of ambient particulate matter (PM), especially fine PM with aerodynamic diameters ≤2.5 μm (PM_2.5_), may increase cardiovascular morbidity by causing hypertension, coronary heart disease, stroke, and diabetes. Besides inflammation and oxidative stress, central nervous system activation could also be responsible. The HPA and sympathoadrenomedullary axis are stimulated upon inhalation of PM_2.5_ (or lower) particles in humans [31,32].

Depression, anxiety, metabolic dysfunction, and obesity are also associated with the potential adverse effects of EDCs on adrenals [33].

There are relatively few studies on the effect of EDCs on the adrenal medulla. DDT-exposed rats tend to have lower blood epinephrine levels than unexposed controls. Additionally, DDT has been found to inhibit tyrosine hydroxylase synthesis and to impact the mitochondrial apparatus of epinephrine-producing cells [3,34].

EDCs may affect adrenal glands, and therefore the adrenal–gonadal system. Early pubarche was observed in boys exposed to phthalates, along with high testosterone and low adrenal androgen levels [35,36]. EDCs may be responsible for testicular dysgenesis syndrome (TDS) in humans. This syndrome is characterized by oligospermia, cryptoorchidism, hypospadias, and testicular carcinoma [37]. Phthalates can disrupt androgen action. Animal studies have shown that male rat fetuses exposed to phthalate causes similar symptoms to TDS. There are several studies that show the relationship between prostate cancer and EDCs. Estrogen receptors are present in prostate tissue and, along with androgen, estrogen has a role in the development of benign prostate hyperplasia and prostate cancer. Pesticide, dioxin, BPA, and alcohol phenol exposure increases prostate cancer incidence [2,38,39,40]. Exposure to EDCs can lead to various problems in the female reproductive system depending on the type of chemical and exposure time. Early puberty, polycystic ovary syndrome (PCOS), premature ovarian failure, and endometriosis are associated with EDCs. In utero exposure to EDCs can play a role in PCOS development in female fetuses. Some PCOS patients have excess adrenal precursor androgens, such as dehydroepiandrosterone (sulfate). High testosterone exposure before birth has linked to increased PCOS risk. In an observational study, females with PCOS had higher serum BPA levels compared to a control group [41–44]. Transgenerational effects of EDCs on adrenal tissue still requires investigation.

## 3. Conclusion

Most EDCs are synthetic industrial chemicals. They can spread through water, soil, and air. Acute exposure to toxic high doses and constant exposure to low doses of EDCs may cause harmful consequences. Endocrine pathways can be affected by EDCs via accumulation in lipophilic tissue. Although the mechanism of action is not fully understood, EDC exposure leads to adrenocortical disruption. The current evidence shows that adrenal glands are common targets for drugs and chemicals and the main mechanism is steroidogenic pathway inhibition. Every step and enzyme in mineralocorticoid or glucocorticoid pathways can be affected by EDCs. In order to avoid the long-term consequences of EDCs, it is crucial to be aware of their potential harmful effects.

## Figures and Tables

**Table:** Examples of chemicals and pharmaceutical medicines that target steroidogenic pathway and cause adrenocortical toxicity.

Compound	Targets in steroidogenic pathway and regions of adrenal cortex
Dichlorodiphenyltrichloroethane (DDT)	Cell atrophy and degeneration in adrenal zona fasciculata ve zona reticularis [1]
Polychlorinated biphenyls (PCB)	CYP17, CYP21, CYP11B1, CYP11B2 and 3-hydroxysteroid dehydrogenase D4,5 isomerase [8]
Tetrabromobisphenol-A	CYP17 [18,33]
2,3,7,8-tetrabromodibenzo-p-dioxin	3-Hydroxysteroid dehydrogenase D4,5 isomerase [8,19 ]
Aminoglutethimide	ACTH receptors, CYP11A1 and CYP11B1 [8,16]
Etomidate	CYP11B1 [8,17]
Flavonoids	CYP21, CYP11B1 [20]
Bromophenols	3-hydroxysteroid dehydrogenase D4,5 isomerase [19]
Cyanoketonea	3-hydroxysteroid dehydrogenase D4,5 isomerase [28]

## References

[1] Timokhina E Yaglov V Nazimova S Dichlorodiphenyltrichloroethane and the Adrenal Gland: From Toxicity to Endocrine Disruption Toxics 2021 9 10 243 10.3390/toxics9100243 34678939 PMC8539486

[2] Gore AC Chappell VA Fenton SE Flaws JA Nadal A EDC-2: The Endocrine Society’s second scientific statement on endocrine-disrupting chemicals Endocrine Reviews 2015 36 6 E1 E150 10.1210/er.2015-1010 26544531 PMC4702494

[3] Egalini F Marinelli L Rossi M Motta G Prencipe N Endocrine disrupting chemicals: effects on pituitary, thyroid and adrenal glands Endocrine 2022 78 3 395 405 10.1007/s12020-022-03076-x 35604630 PMC9637063

[4] Zou E Invisible endocrine disruption and its mechanisms: A current review General and Comparative Endocrinology 2020 293 113470 10.1016/j.ygcen.2020.113470 32234298

[5] Ahn C Jeung EB Endocrine-Disrupting Chemicals and Disease Endpoints International Journal of Molecular Sciences 2023 24 6 5342 10.3390/ijms24065342 36982431 PMC10049097

[6] Vinnars M Bixo M Damdimopoulou P Pregnancy-related maternal physiological adaptations and fetal chemical exposure Molecular and Cellular Endocrinolog 2023 578 112064 10.1016/j.mce.2023.112064 37683908

[7] Auchus R Pandey C The Adrenal Cortex Melmed S Auchus R Goldfine A Rosen C Kopp P Williams Textbook of Endocrinology 15th ed Philadelphia, PA 2024 476

[8] Harvey P Adrenocortical endocrine disruption Journal of Steroid Biochemistry and Molecular Biology 2016 155 199 206 10.1016/j.jsbmb.2014.10.009 25460300

[9] Hontela A Vijayan MM Adrenocortical toxicology in fishes Harvey PW Everett DJ Springall CJ Adrenal Toxicology 1st ed Informa Healthcare New York 2009 233 256

[10] Schwake LH Smith CR Townsend F Wells R Hart L Health of common bottlenose dolphins (Tursiops truncatus) in Barataria Bay Louisiana, following the Deepwater Horizon oil spill Environmental Science and Technology 2014 48 1 93 103 10.1021/es403610f 24350796

[11] Fu X Blaydes B Weis C Latendresse J Muskhelishvili L Effects of dietary soy and estrous cycle on adrenal cytochrome P450 1B1 expression and DMBA metabolism in adrenal glands and livers in female Sprague–Dawley rats Chemico-Biological Interactions 2003 146 3 273 284 10.1016/j.cbi.2003.09.004 14642739

[12] Lund BO Bergman A Brandt I Metabolic activation and toxicity of a DDT-metabolite, 3-methylsulphonyl-DDE, in the adrenal Zona fasciculata in mice Chemico-Biological Interactions 1988 65 1 25 40 10.1016/0009-2797(88)90028-2 3345572

[13] Ahmed AE Jacob S Ghanayem B Comparative disposition of acrylonitrile and metha-crylonitrile: quantitative whole-body autoradiographic studies in rats Fundamental and Applied Toxicology 1996 33 1 49 59 10.1006/faat.1996.0142 8812221

[14] Brandt I Bergman A PCB methyl sulphones and related compounds: identification of target cells and tissues in different species Chemosphere 1987 16 1671 1676 10.1016/0045-6535(87)90147-0

[15] Meli R Monnolo A Annunziata C Pirozzi C Ferrante MC Oxidative stress and BPA toxicity: an antioxidant approach for male and female reproductive dysfunction Antioxidants 2020 9 5 405 10.3390/antiox9050405 32397641 PMC7278868

[16] Fassnacht M Beuschlein F Vay S Mora P Allolio B Aminoglutethimide suppresses adrenocorticotrophin receptor expression in the NCI-h295 adrenocortical tumor cell line The Journal of Endocrinology 1998 159 1 35 42 10.1677/joe.0.1590035 9795339

[17] Hille U Zimmer C Vock C Hartmann R First Selective CYP11B1 Inhibitors for the Treatment of Cortisol-Dependent Diseases ACS Medicinal Chemistry Letters 2011 2 1 2 6 10.1021/ml100071j 24900247 PMC4018151

[18] Knížatová N Greifová H Tokárová K Jambor T Binkowski ŁJ Assessment of the effective impact of bisphenols on mitochondrial activity, viability and steroidogenesis in a dose-dependency in human adrenocortical carcinoma cells Processes 2021 9 8 1471 10.3390/pr9081471

[19] Ding l Murphy MB He Y Xu Y Yeung L Effects of brominated flame retardants and brominated dioxins on steroidogenesis in H295R human adrenocortical carcinoma cell line Environmental Toxicology and Chemistry 2007 26 26 764 772 10.1897/06-388r1.1 17447562

[20] Ohno S Shinoda S Toyoshima S Nakazawa H Makino T Effects of flavonoid phytochemicals on cortisol production and on activities of steroidogenic enzymes in human adrenocortical H295R cells The Journal of Steroid Biochemistry and Molecular Biology 2002 80 3 355 363 10.1016/s0960-0760(02)00021-3 11948020

[21] Nowak K Wrona W Górska M Jabłońska E Parabens and their effects on the endocrine system Molecular and Cellular Endocrinology 2018 474 238 251 10.1016/j.mce.2018.03.014 29596967

[22] Taxvig C Vinggaard AM Hass U Axelstad M Boberg J Do parabens have the ability to interfere with steroidogenesis? Toxicological Sciences 2008 106 1 206 213 10.1093/toxsci/kfn148 18648085

[23] Sanderson JT Boerma J Lansbergen GW Berg M Induction and inhibition of aromatase (CYP19) activity by various classes of pesticides in H295R human adrenocortical carcinoma cells Toxicology and Applied Pharmacology 2002 182 1 44 54 10.1006/taap.2002.9420 12127262

[24] Gracia T Hilscherova K Jones PD Newsted J Higley E Modulation of steroidogenic gene expression and hormone production of H295R cells by pharmaceuticals and other environmentally active compounds Toxicology and Applied Pharmacology 2007 225 2 142 153 10.1016/j.taap.2007.07.013 17822730

[25] Fommei E Turci R Ripoli A Balzan S Bianchi F Evidence for persistent organochlorine pollutants in the human adrenal cortex Journal of Applied Toxicology 2017 37 9 1091 1097 10.1002/jat.3460 28332723

[26] Nowak K Szklarz M Szychlinska M Matuszewski W Stankiewicz E A Brief Look at Hashimoto’s Disease, Adrenal Incidentalomas, Obesity and Insulin Resistance—Could Endocrine Disruptors Be the Other Side of the Same Coin? Medicina 2023 59 7 1234 10.3390/medicina59071234 37512046 PMC10385892

[27] Ribelin WE The effects of drugs and chemicals upon the structure of the adrenal gland Fundamental and Applied Toxicology 1984 4 1 105 119 10.1016/0272-0590(84)90224-0 6692999

[28] Akana SF Shinsako J Dallman MF Drug-induced adrenal hypertrophy provides evidence for reset in the adrenocortical system Endocrinology 1983 113 2232 2237 10.1210/endo-113-6-2232 6315345

[29] Panagiotidou E Zerva S Mitsiou DJ Alexis MN Kitraki E Perinatal exposure to low-dose bisphenol A affects the neuroendocrine stress response in rats Journal of Endocrinology 2014 220 3 207 218 10.1530/JOE-13-0416 24323913

[30] Lee S Martinez-Arguelles DB Campioli E Papadopoulos V Fetal Exposure to Low Levels of the Plasticizer DEHP Predisposes the Adult Male Adrenal Gland to Endocrine Disruption Endocrinology 2017 158 2 304 318 10.1210/en.2016-1604 27849367

[31] Li H Cai J Chen R Zhao Z Ying Z Particulate Matter Exposure and Stress Hormone Levels: A Randomized, Double-Blind, Crossover Trial of Air Purification Circulation 2017 136 7 618 627 28808144 10.1161/CIRCULATIONAHA.116.026796

[32] Toledo-Corral CM Alderete TL Herting MM Habre R Peterson AK Ambient air pollutants are associated with morning serum cortisol in overweight and obese Latino youth in Los Angeles Environmental Health 2021 20 39 10.1186/s12940-021-00713-2 33832509 PMC8034084

[33] Pötzl B Kurzinger L Stopper H Fassnacht M Kurlbaum M Endocrine Disruptors: Focus on the Adrenal Cortex Hormone and Metabolic Research 2024 56 1 78 90 10.1055/a-2198-9307 37884032 PMC10764154

[34] Yaglova N Obernikhin S Tsomartova D Yaglov V Nazimova S Impact of Prenatal and Postnatal Exposure to Endocrine Disrupter DDT on Adrenal Medulla Function International Journal of Molecular Sciences 2022 23 9 4912 10.3390/ijms23094912 35563302 PMC9101091

[35] Mouritsen A Frederiksen H Sørensen K Aksglaede L Hagen C Urinary Phthalates from 168 Girls and Boys Measured Twice a Year during a 5-Year Period: Associations with Adrenal Androgen Levels and Puberty The Journal of Clinical Endocrinology and Metabolism 2013 98 9 3755 3764 10.1210/jc.2013-1284 23824423

[36] Predieri B Iughetti L Bernasconi S Strret M Endocrine Disrupting Chemicals’ Effects in Children: What We Know and What We Need to Learn? International Journal of Molecular Science 2022 23 19 11899 10.3390/ijms231911899 PMC9570268 36233201

[37] Xin Hu G Quan Lian Q Shan Ge R Hardy D Kun Li X Phthalate-induced testicular dysgenesis syndrome: Leydig cell influence Trends in Endocrinology Metabolism 2009 20 3 139 145 10.1016/j.tem.2008.12.001 19278865 PMC2718776

[38] Ho SM Tang WY Belmonte de Frausto J Prins GS Developmental exposure to estradiol and bisphenol A increases susceptibility to prostate carcinogenesis and epigenetically regulates phosphodiesterase type 4 variant 4 Cancer Research 2006 66 11 5624 5632 10.1158/0008-5472.CAN-06-0516 16740699 PMC2276876

[39] Vom Saal FS Timms BG Montano MM Palanza P Thayer K Prostate enlargement in mice due to fetal exposure to low doses of estradiol or diethylstilbestrol and opposite effects at high doses Proceedings of the National Academy of Sciences 1997 94 5 2056 2061 10.1073/pnas.94.5.2056 PMC20042 9050904

[40] Leng L Chen X Li PC Luo YX Tang JN 2,3,7,8-Tetrachlorodibezo-p-dioxin exposure and prostate cancer: a meta-analysis of cohort studies Public Health 2014 128 3 207 213 10.1016/j.puhe.2013.10.006 24461260

[41] Peretz J Vrooman L Ricke WA Hunt P Erlich S Bisphenol A and reproductive health: update of experimental and human evidence, 2007–2013 Environmental Health Perspectives 2014 122 8 775 786 10.1289/ehp.1307728 24896072 PMC4123031

[42] Caserta D Di Segni N Mallozzi M Giovanale V Mantovani A Bisphenol A and the female reproductive tract: an overview of recent laboratory evidence and epidemiological studies Reproductive Biology Endocrinology 2014 12 37 10.1186/1477-7827-12-37 24886252 PMC4019948

[43] Abbott DH Nicol LE Levine JE Xu N Goodarzi MO Nonhuman primate models of polycystic ovary syndrome Molecular and Cellular Endocrinology 2013 373 1–2 21 28 10.1016/j.mce.2013.01.013 23370180 PMC3683573

[44] Kandaraki E Chatzigeorgiou A Livadas S Palioura E Economou F Endocrine disruptors and polycystic ovary syndrome (PCOS): elevated serum levels of bisphenol A in women with PCOS The Journal of Clinical Endocrinology and Metabolism 2011 96 3 E480 E484 10.1210/jc.2010-1658 21193545

